# Practice in Philadelphia

**Published:** 1852-12

**Authors:** 


*Practice in Philadelphia.*—From the Philadelphia Medical and surgical Journal, the following extract is taken: "Two-thirds of the practice of medicine in Philadelphia, even among those who pay for everything else, is done gratis; and not unfrequently insult is added to injury. This is due to the crowded state of the profession, and to the *mock charity* inculcated by many of the public teachers. The average fee for medical attendance, *paid*, does not exceed *twenty five cents* a visit."

Perhaps some one, more familiar with the phases of professional income in Boston than ourselves, may favor us with a statement of the profits of practice here. Some certainly have large incomes; while others, whose merits and qualifications cannot be questioned, have none at all. *Boston Med. Jour.*

